# Allelopathic Effect of *Humulus scandens* Stem and Leaf Extract on *Solidago canadensis*

**DOI:** 10.3390/life16091452

**Published:** 2026-08-31

**Authors:** Bo Xie, Yiming Lai, Jun Wang, Huijuan Cao, Sheng Qiang, Zhen Zhang, Zheng Zhang, Ming Tang, Chunhuo Zhou, Zunkang Zhao

**Affiliations:** 1 Key Laboratory of Agricultural Resources and Ecology in Poyang Lake Watershed of Ministry of Agriculture and Rural Affairs in China, College of Land Resource and Environment, Jiangxi Agricultural University, Nanchang 330045, China; bobo25330@163.com (B.X.);; 2 Xingguo County Xingjiang Township People’s Government, Ganzhou 342403, China; 3 Technology and Ecology Division, Department of Agriculture and Rural Affairs of Jiangxi Province, Nanchang 330046, China; 4 Weed Research Laboratory, Nanjing Agricultural University, Nanjing 210095, China; 5 College of Resources and Environment, Anhui Agricultural University, Hefei 230036, China; 6 Jiangxi Provincial Key Laboratory of Conservation Biology, Jiangxi Agricultural University, Nanchang 330045, China

**Keywords:** *Humulus scandens*, *Solidago canadensis*, allelopathy, ethyl acetate, metabolomics

## Abstract

*Solidago canadensis* is one of the most destructive invasive plants in China, and current physical and chemical control methods are often costly, inefficient, and ecologically risky, highlighting the urgent demand for green and efficient ecological alternatives. The native vine *Humulus scandens* exhibits strong allelopathic potential. In this study, allelochemicals from its leaves and stems were extracted using petroleum ether, ethyl acetate, n-butanol, and n-hexane. Their allelopathic effects on *S. canadensis* were systematically evaluated through seed germination, seedling growth, and rhizome propagation assays, combined with widely targeted metabolomics. Leaf extracts showed markedly stronger allelopathic inhibition than stem extracts. Among all fractions, the ethyl acetate extract exerted the most potent suppression on seed germination, significantly reducing germination rate and root elongation in a concentration-dependent manner. At 100 g·L^−1^, leaf extracts—especially the ethyl acetate phase—significantly decreased belowground fresh weight, rhizome number, and new seedling emergence. The ethyl acetate extract also significantly elevated root antioxidant enzyme activities at 25–100 g·L^−1^, indicating induced oxidative stress. UPLC-MS/MS-based metabolomics identified 186 differential metabolites among extracts. Ten flavonoids were significantly upregulated in the ethyl acetate extract and were predominantly enriched in the kaempferol aglycone biosynthesis I pathway (MetMap 113) and the flavonoid biosynthesis pathway (ko00941), suggesting that these compounds may play a key role in the observed allelopathic inhibition. Collectively, the ethyl acetate extract of *H. scandens* leaves effectively suppresses *S. canadensis* growth by inhibiting seed germination and rhizome tillering and by inducing oxidative stress, probably with the 10 candidate flavonoid metabolites, highlighting its strong potential as a biological control agent against this invasive weed.

## 1. Introduction

The spread of invasive alien plant species has emerged as a global environmental challenge, posing significant threats to plant biodiversity and ecosystem stability [1,2]. In the absence of natural enemies or competitive pressures, invasive species can efficiently exploit available resources, leading to severe disruptions of local plant communities and alterations to the physical environment. Such invasions can not only modify the structure and functional dynamics of ecosystems but may also contribute to the local or even global extinction of indigenous species [3,4].

*Solidago canadensis* L., a perennial herb indigenous to North America, has become a globally invasive plant species [5,6]. Due to its rapid establishment across diverse habitats and the formation of monodominant communities, it strongly suppresses the growth of native plant species, significantly impacting local biodiversity and the stability of natural ecosystems. It is now recognized as one of the most widespread and ecologically damaging invasive plants in China [7,8]. In recent years, the management of *Solidago canadensis* has primarily relied on mechanical removal and the application of chemical herbicides. However, these approaches are associated with high costs, limited long-term efficacy, and potential unintended consequences such as the acceleration of invasion spread and the development of herbicide-resistant weed populations, all of which compromise control effectiveness [9,10,11]. Therefore, the development of more efficient and sustainable control strategies has become a focal point of current research. In the field of biological control, efforts have mainly concentrated on the identification and application of pathogenic microorganisms, with particular emphasis on pathogenic fungi. For example, the toxin produced by *Sclerotium rolfsii*, a fungal pathogen, significantly inhibits the photosynthetic capacity of *Solidago canadensis* by damaging leaf cell structures, thereby limiting its growth and expansion. *Rhizoctonia solani* has been shown to induce white mold disease in this invasive species. Additionally, the combined use of the bioherbicide SC64 and tillage practices has been found to effectively suppress the regenerative potential of *Solidago canadensis* [12,13,14]. Nevertheless, fungal-based biological control methods remain at the experimental stage, lacking fully developed technical frameworks and consistent field performance, and thus have not yet been widely implemented.

Allelopathy plays a crucial role in both agricultural and natural ecosystems and has garnered increasing attention as a promising alternative strategy for managing invasive plant species [15,16]. Compared to conventional physical or chemical control methods, allelochemicals are naturally produced and biodegradable, resulting in minimal environmental impact, which makes them a more sustainable control option. Allelopathy refers to the ecological phenomenon in which plants release secondary metabolites, known as allelochemicals, into the environment, thereby exerting direct or indirect inhibitory effects on the growth of neighboring plants [17,18]. These allelochemicals can not only directly suppress the growth of target species by interfering with key physiological processes such as photosynthesis, respiration, enzyme synthesis, and metabolic activities, but also indirectly influence plant community composition by modifying soil nutrient dynamics and affecting the activity of beneficial soil microorganisms [19,20]. The rhizosphere biochemical processes mediated by allelopathic interactions may promote coevolution within native habitats, thereby shaping species coexistence mechanisms and the structural evolution of plant communities, ultimately contributing to effective invasive plant control [21]. In recent years, ecological management strategies based on alternative planting have shown initial success in the biological control of invasive species. For example, in the management of invasive plants such as *Alternanthera philoxeroides* [22], *Ambrosia trifida* [23], and *Sphagneticola trilobata* [24], native plant species with competitive advantages have been utilized to occupy the ecological niches of invasive species, effectively inhibiting their growth and spread. Furthermore, various cover crops have demonstrated strong allelopathic potential, enabling effective weed suppression without the use of synthetic herbicides [25]. Utilizing such plant-based strategies not only reduces reliance on chemical inputs in agricultural systems but also mitigates adverse environmental consequences such as bioaccumulation and biomagnification [26]. However, systematic research on the screening of alternative plant species, mechanistic studies, and field validation for the ecological control of *Solidago canadensis* remains limited, and publicly available reports on this topic are still scarce.

Previous field surveys have indicated that the native plant species *Humulus scandens* exerts a notable inhibitory effect on *Solidago canadensis*, exhibiting a distinct competitive ecological niche. Research has demonstrated that *Humulus scandens* displays varying levels of allelopathic activity, which can effectively suppress the photosynthetic capacity of *Eichhornia crassipes*. Moreover, its aqueous extract has been shown to significantly inhibit the vegetative growth of this invasive species [27,28]. *Humulus scandens* possesses strong allelopathic potential, and its aqueous extract has been confirmed to markedly hinder seed germination and seedling development in a variety of plant species [29,30]. Using GC-MS analysis, researchers have preliminarily identified the presence of allelochemicals such as fatty acids, steroids, and terpenoids in *Humulus scandens*, which are commonly reported bioactive constituents in existing literature [31]. Therefore, it is hypothesized that *Humulus scandens* may interfere with the germination and growth of *Solidago canadensis* through the release of these allelochemicals, thereby demonstrating considerable potential for ecological control. However, current studies on the allelopathic effects of *Humulus scandens* have primarily focused on observable physiological and biochemical responses, as well as early developmental stages such as seed germination and seedling growth, while the underlying molecular mechanisms remain largely unexplored.

Metabolomics approaches have been extensively employed to investigate the allelopathic mechanisms of invasive alien plant species [32,33,34]. Through the integration of metabolomic data derived from diverse samples, it is feasible to effectively characterize the distribution patterns of differential metabolites across samples, offering valuable insights into the underlying mechanisms of allelochemical action in plants. Notably, metabolomics analysis facilitates the identification of differential metabolic features that are closely associated with gene expression profiles, thereby elucidating key functional genes, signaling pathways, and their regulatory networks in plant systems [35,36,37]. UPLC-MS/MS technology, as a highly efficient and reliable analytical tool in metabolomics, has emerged as a primary method for the detection and profiling of plant metabolites. This technique not only enables precise identification and quantification of metabolites but also provides opportunities for in-depth understanding of the molecular mechanisms and stress response pathways involved in plant metabolic regulation [38]. Utilizing this approach, researchers are able to investigate the dynamic alterations in plant metabolic networks under varying environmental conditions or experimental treatments, thereby further elucidating how plants modulate their metabolic profiles to adapt to external stressors. Therefore, to achieve a comprehensive understanding of the roles and mechanisms of diverse metabolites, future research should prioritize systematic and large-scale investigations that integrate metabolite identification with quantitative analysis, thereby advancing the in-depth exploration of allelopathy and its regulatory mechanisms in plant systems.

To clarify the allelopathic mechanisms by which *Humulus scandens* inhibits the invasive weed *Solidago canadensis*, this study was conducted to (i) evaluate the allelopathic potential of different *H. scandens* tissues and extraction solvents, (ii) characterize the physiological and biochemical responses of *S. canadensis*, and (iii) identify key allelochemicals and their metabolic pathways using widely targeted metabolomics. The findings are expected to provide a scientific basis for the biological control of *S. canadensis* and the utilization of allelopathic resources.

## 2. Materials and Methods

### 2.1. Plant Materials

*Humulus scandens* and *Solidago canadensis* plants were collected from a suburban area of Nanchang, Jiangxi Province (115.834451° E, 28.759639° N). After removing soil, plants were separated into stems and leaves, air-dried to constant weight, ground, and sieved through 40-mesh. The powder was stored in sealed bags. For *S. canadensis*, roots were washed, and aboveground parts were trimmed, leaving 5 cm stem segments for pot experiments. *S. canadensis* seeds were collected from mature plants at the same site.

### 2.2. Extract Solution Preparation

Thirty grams of stem and leaf powder was extracted using 150 mL of petroleum ether, ethyl acetate, n-butanol, and n-hexane, all of which were of chromatographic grade (Aladdin Biochemical Technology Co., Ltd., Shanghai, China). The solid–liquid ratio was set to 1:5 (g/mL). The mixture was subjected to shaking extraction at 100 r/min for 24 h at 25 °C, followed by filtration. The residual solid was re-extracted under identical conditions. The combined filtrates were concentrated via rotary evaporation (Shanghai Yarong Biochemical Instrument Factory, Shanghai, China), with parameters set as follows: petroleum ether: 45 °C, 50 r/min; ethyl acetate: 40 °C, 50 r/min; n-butanol: 60 °C, 60 r/min; n-hexane: 50 °C, 50 r/min. The concentrated extracts were dissolved in distilled water via ultrasonic treatment, and solutions with mass concentrations of 0.025, 0.050, 0.100, 0.150, and 0.200 g·mL^−1^ were prepared, respectively, labeled as PE, EA, n-BuOH, and n-H, and stored at 4 °C. The control group adopted the corresponding pure extraction agent and distilled water system, with the same volume and rotary evaporation concentration treatment as the sample solutions [39].

### 2.3. Assessment of the Allelopathic Activity of Humulus scandens Stem and Leaf Extracts

Plump *S. canadensis* seeds were surface-sterilized (75% ethanol, 30 s; 2% sodium hypochlorite, 10 min), rinsed thoroughly, and germinated on two layers of filter paper in 90 mm Petri dishes [40]. Each dish received 30 seeds and 5 mL of extract solution or distilled water (control), with three replicates. Dishes were incubated at 26 ± 1 °C, 80% relative humidity, under a 12 h light/12 h dark photoperiod (18,000 lux) (Bikeman Biotechnology Co., Hunan, China). Moisture was maintained by adding 2 mL of the corresponding solution twice daily. Germination [41] (radicle length > half seed length) was recorded daily until no germination occurred for two consecutive days. After 10 days, shoot and root lengths of 10 randomly selected seedlings (or all if fewer germinated) were measured with a vernier caliper [42]. Germination rate (GR) [43] and germination index (GI) [44] were calculated as:

(1) $$Germination\;rate\left(GR\right)=\frac{Number\;of\;Germinated\;Seeds}{Total\;Number\;of\;Tested\;Seeds}\times 100\%$$

(2) $$Germination\;index\;(GI)=\sum \frac{Gt(Number\;of\;Germinated\;Seeds\;Withint\;Days)}{Dt(Corresponding\;Germination\;Days)}$$

Relative inhibition effect index: This index is based on the comparison of fresh weight changes (*ΔW* = post-treatment weight − pre-treatment weight) with the control group. It reflects the relative degree of the inhibitory effect compared to the fresh weight gain of the control group. The calculation formula is as follows:

(3) $$Relative\;inhibitory\;effect\;index\;(\%)=\frac{\Delta Wcontrol-\Delta Wtreatment}{\Delta Wcontrol}$$

### 2.4. Growth Analysis of Solidago canadensis Plants Treated with Humulus scandens Leaf Extract at the Root Level

In June 2024, 375 uniform *S. canadensis* plants were collected; roots were washed, blotted dry, and weighed. Shoots were cut to a 5 cm stem base. Plants were potted individually (13 cm height × 12 cm diameter) in a 3:1 (*v*/*v*) mixture of nutrient soil and vermiculite, and watered regularly for three days to ensure establishment. Extracts (PE, EA, BH, n-H) were prepared as described in Section 2.2 and diluted to 25, 50, 100, 150, and 200 g·L^−1^. The mixed system of the purified extractants obtained by rotary evaporation and distilled water was used as the control. Starting on day 5 after initial watering, roots were irrigated with 50 mL of the respective solution every five days for a total of six applications, with five plants per concentration and three replicates. Thirty days after the first treatment, plants were harvested. Root fresh weight change (*ΔW*), number of rhizomes, and new seedlings were recorded. Activities of superoxide dismutase (SOD), peroxidase (POD), catalase (CAT), and malondialdehyde (MDA) content were measured using assay kits (Beijing Solarbio Science & Technology Co., Ltd., Beijing, China; http://www.solarbio.com) following the manufacturer’s instructions.

### 2.5. Assessment of Allelopathic Effects

The allelopathic response index (RI) was employed to determine the nature of allelopathic interactions, while the Synthetical effect of allelopathy index (SE) was analyzed to evaluate the overall impact of various treatments on the seeds and root segments of *Solidago canadensis*. The specific determination procedures are described as follows:

(4) $$RI=\left\{\begin{array}{c} 1-\frac{C}{T}\;\;\left(T\geq C\right)\\ \frac{T}{C}-1\;\;\left(T<C\right) \end{array}\right.$$

In this context, T denotes the growth response of the test material subjected to the extract solution, whereas C denotes the growth response of the test material treated with distilled water (control group). A positive RI value indicates a stimulatory effect of the extract solution on seedling growth, while a negative RI value reflects an inhibitory effect [45].

(5) $$SE=\frac{{RI}_{1}+{RI}_{2}+\cdots +{RI}_{n}}{n}$$

In this context, *n* denotes the number of allelochemical types, while SE represents the overall intensity of allelopathic effects. SE is utilized to assess the average allelopathic impact across multiple allelochemicals. A greater absolute value of SE indicates a stronger overall allelopathic effect.

### 2.6. UPLC-MS/MS Analysis

Each extract obtained in Section 2.2 was concentrated, dissolved in 10 mL of the corresponding chromatographic grade solvent (petroleum ether, ethyl acetate, n-butanol, or n-hexane), and dried under nitrogen. A 20 mg portion of each dried sample was extracted with 1 mL of 70% methanol containing internal standard (1:50, *v*/*v*), vortexed for 10 min, sonicated in an ice-water bath for 10 min, and centrifuged for 3 min. The supernatant was filtered through a 0.22 μm microporous membrane and stored at −20 °C until analysis. Metabolome analysis was performed by Wuhan Maiwei Biotechnology Co., Ltd.

Sample extracts were analyzed on a UPLC-ESI-MS/MS system consisting of an ExionLC™ AD UPLC coupled to a QTRAP tandem mass spectrometer (SCIEX, Framingham, MA, USA; https://www.sciex.com/). UPLC conditions: Agilent SB-C18 column (1.8 μm, 2.1 mm × 100 mm); mobile phase A, water with 0.1% formic acid, and B, acetonitrile with 0.1% formic acid. The gradient program started at 95% A/5% B, changed linearly to 5% A/95% B over 9 min, was held for 1 min, then returned to 95% A/5% B in 1.1 min and held for 2.9 min. Flow rate, 0.35 mL/min; column temperature, 40 °C; injection volume, 2 μL. The ESI source parameters were: temperature 500 °C; ion spray voltage 5500 V (positive)/−4500 V (negative); ion source gas I, gas II, and curtain gas at 50, 60, and 25 psi, respectively; collision-activated dissociation (CAD) high. MRM scans were acquired with medium collision gas. Declustering potential (DP) and collision energy (CE) were optimized for individual MRM transitions, and a specific set of transitions was monitored for each period according to the eluted metabolites.

### 2.7. Multivariate Analysis of Metabolite Profiles

Qualitative analysis was performed using secondary mass spectrometry data and the in-house Metware Database (MWDB). Quantitative analysis was conducted in MRM mode, with chromatographic peak areas integrated and normalized across samples. Mass spectrometry data were processed with Analyst 1.6.3 software. Unsupervised PCA was performed using the prcomp function in R with unit variance scaling. Hierarchical cluster analysis (HCA) and Pearson correlation coefficients (PCC) were calculated and visualized using the R package ComplexHeatmap. HCA results for samples and metabolites were presented as heatmaps with dendrograms, while PCC results were presented as heatmaps only. For HCA, normalized signal intensities (unit variance scaled) were displayed as a color spectrum.

### 2.8. Identification and Characterization of Differentially Regulated Metabolites

For two-group analysis, differential metabolites were determined by VIP (VIP > 1) and absolute Log_2_FC (|Log_2_FC| ≥ 1.0). VIP values were extracted from OPLS-DA results, which also contain score plots and permutation plots, generated using R package MetaboAnalystR. The data was log-transformed (log_2_) and subjected to mean centering before OPLS-DA. In order to avoid overfitting, a permutation test (200 permutations) was performed. Identified metabolites were annotated using the KEGG Compound database (http://www.kegg.jp/kegg/compound/) (accessed on 16 July 2026); annotated metabolites were then mapped to the KEGG Pathway database (http://www.kegg.jp/kegg/pathway.html) (accessed on 25 October 2024).

### 2.9. Statistical Analysis

Data organization and analysis were carried out using Microsoft Excel 2019. One-way analysis of variance (ANOVA) and Duncan’s Multiple Range Test were conducted using IBM SPSS Statistics 27.0 to evaluate the statistical significance and differences among experimental data. The level of statistical significance was set at *p* < 0.01. Data are presented as mean ± standard error (*n* ≥ 3 biological replicates). Prior to performing ANOVA, the assumptions of homogeneity of variance and normality were assessed. Graphical representations were generated using Origin 2021 software, with data displayed as “mean ± standard error”.

## 3. Results

### 3.1. Effects of Various Types and Concentrations of Humulus scandens Extracts on the Germination and Growth of Solidago canadensis Seeds

**Figure 1:**
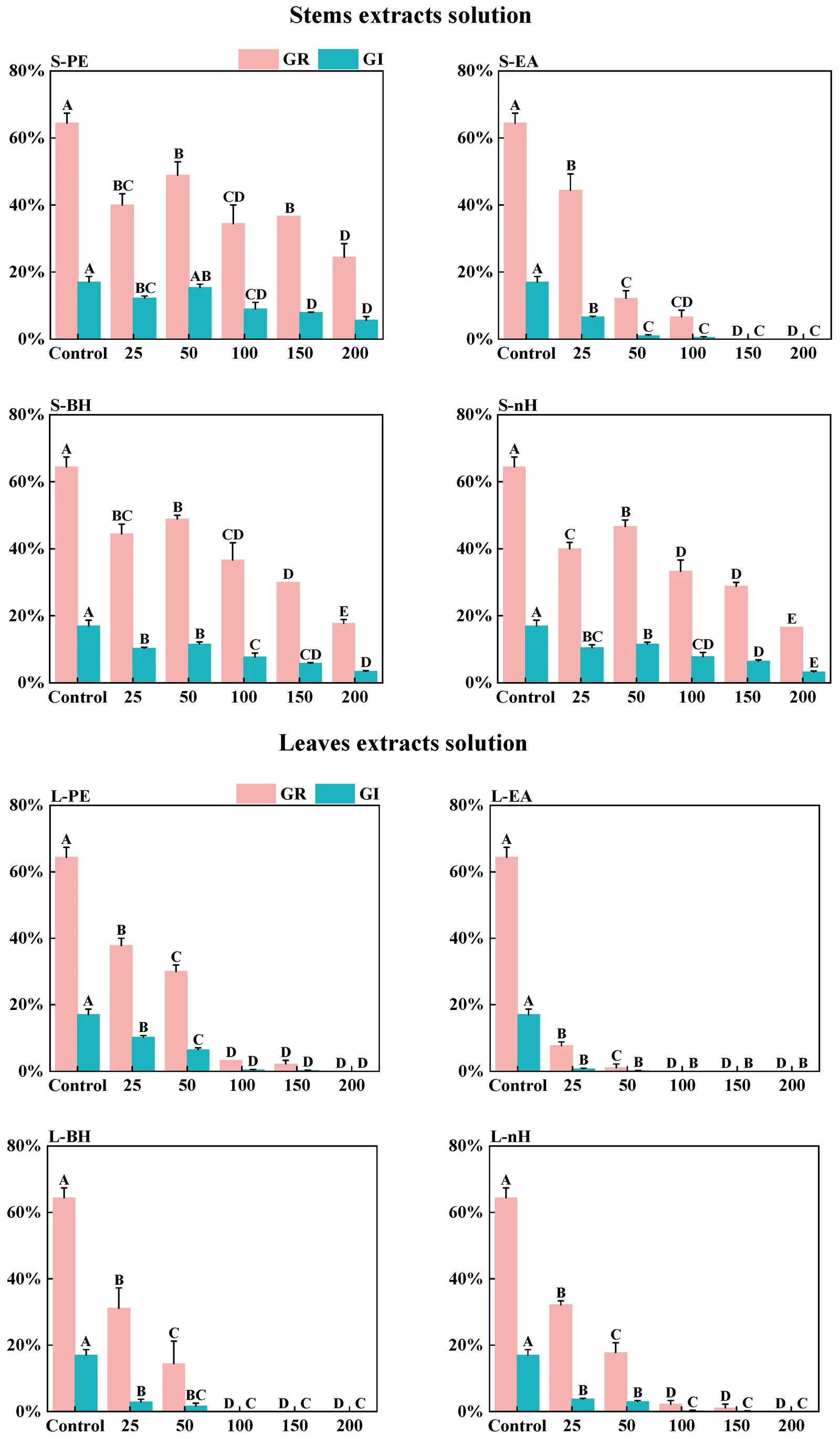
Effects of *Humulus scandens* extracts on the germination of *Solidago canadensis* seeds. Different capital letters indicate statistically significant differences among concentration treatments (*p* < 0.01). The concentration unit for treatment groups is milligrams per milliliter (g·L^−1^). Data are presented as mean ± standard error (*n* ≥ 3 biological replicates). (S-PE) Stem-derived petroleum ether extract. (S-EA) Stem-derived ethyl acetate extract. (S-BH) Stem-derived n-butanol extract. (S-nH) Stem-derived n-hexane extract. (L-PE) Leaf-derived petroleum ether extract. (L-EA) Leaf-derived ethyl acetate extract. (L-BH) Leaf-derived n-butanol extract. (L-nH) Leaf-derived n-hexane extract. (GR) Germination rate. (GI) Germination index.

Extracts from *H. scandens* stems and leaves, prepared with different solvents and concentrations, significantly inhibited seed germination and seedling growth of *S. canadensis* (Figure 1). Leaf extracts showed a concentration-dependent inhibition, reaching 100% at 200 g·L^−1^; ethyl acetate extract was the most potent among all solvents. Stem extracts were generally weaker, with a slight stimulation of germination at 50 g·L^−1^ that remained below control levels. Germination index decreased with increasing concentration for all extracts, approaching zero at 200 g·L^−1^ for leaf extracts.

**Figure 2:**
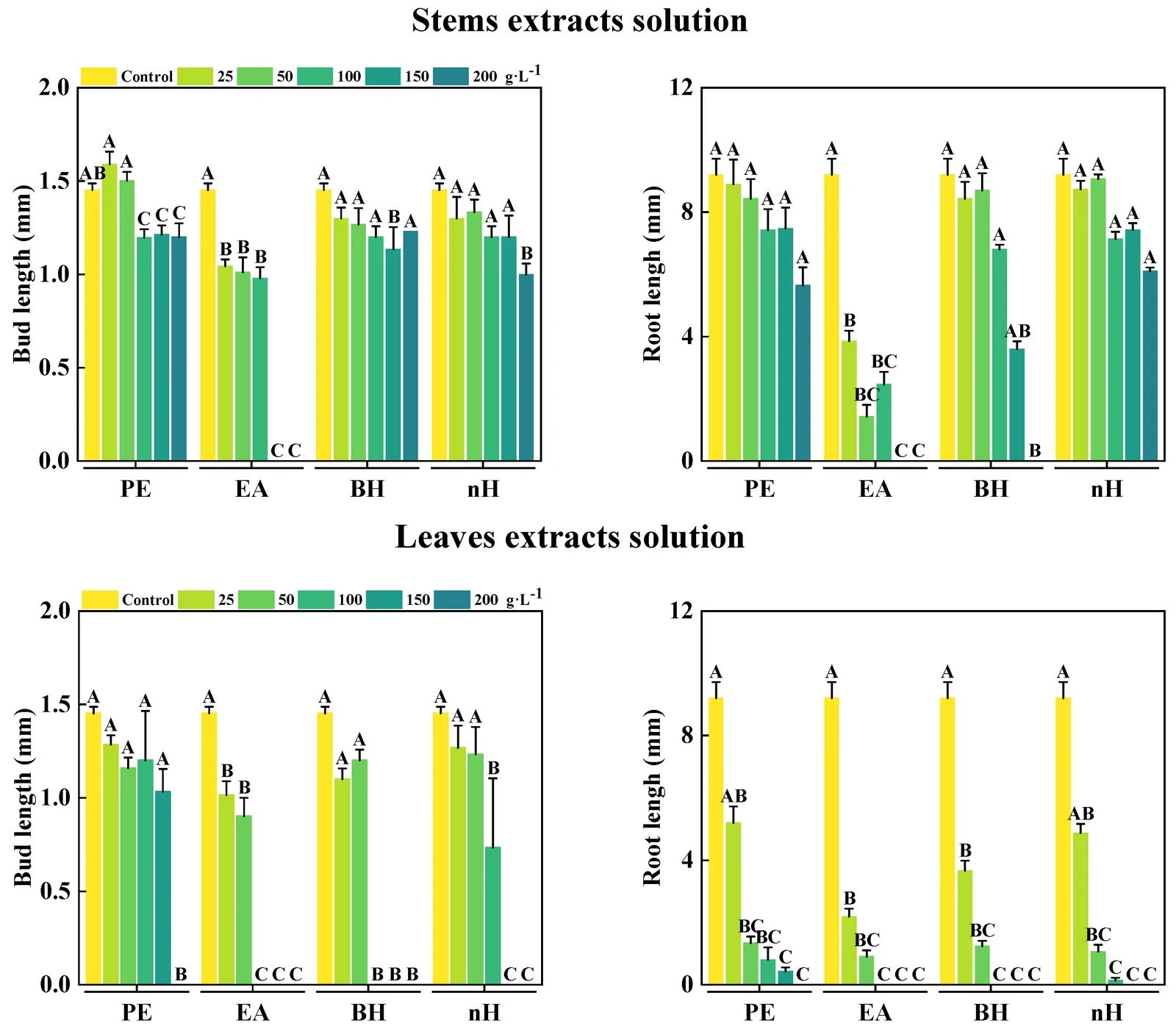
Effects of *Humulus scandens* Extracts on the Growth of *Solidago canadensis* Seedlings. Different capital letters indicate statistically significant differences among concentration treatments (*p* < 0.01). The concentration unit for treatment groups is milligrams per milliliter (g·L^−1^). (PE) petroleum ether extract; (EA) ethyl acetate extract; (BH) n-butanol extract; (nH) n-hexane extract.

During seedling growth, root length was more affected than shoot length (Figure 2). Among stem extracts, only ethyl acetate caused significant root inhibition; the other solvents showed no significant effect up to 100 g·L^−1^. Leaf extracts inhibited root elongation more strongly than stem extracts, with inhibition rates of 43.6–60.1% at 25 g·L^−1^. At 50 g·L^−1^, all four leaf extracts suppressed root growth similarly, and complete inhibition was achieved at 100 g·L^−1^ for ethyl acetate, 150 g·L^−1^ for n-butanol, and 200 g·L^−1^ for petroleum ether and n-hexane.

### 3.2. Assessment of the Allelopathic Effects of Humulus scandens on Solidago canadensis Seeds Germination and Growth

The allelopathic effects of extracts obtained from different parts of *Humulus scandens*, using various solvents and concentrations, demonstrated a consistent trend of increased inhibition with higher extract concentrations on the germination rate, germination index, and root length of *Solidago canadensis* seeds (Table 1). Leaf extracts exhibited a stronger inhibitory effect compared to stem extracts. The inhibitory efficacy of the different solvent extracts followed the order: ethyl acetate > n-butanol > n-hexane > petroleum ether. Ethyl acetate extracts displayed pronounced inhibitory activity across all tested concentrations. Notably, at 200 g·L^−1^, all morphological parameters were completely suppressed, with an inhibition rate of 100% and an allelopathic response index of −1.00. The inhibitory effect of petroleum ether extracts increased progressively with concentration; at 25 g·L^−1^, the inhibition of germination rate and shoot length was minimal, but became significantly stronger at higher concentrations. In contrast, n-butanol and n-hexane extracts exhibited only weak allelopathic effects at lower concentrations (25 g·L^−1^ and 50 g·L^−1^), indicating limited inhibitory potential. The responses of different morphological indicators to stem and leaf extracts varied. Under ethyl acetate and n-butanol treatments, leaf extracts showed stronger inhibition, particularly at 200 g·L^−1^, where the suppression of germination rate and root length was most pronounced. Conversely, under petroleum ether and n-hexane treatments, the allelopathic effects on leaf extracts were less evident.

### 3.3. Effects of Different Types of Humulus scandens Leaf Extracts on Morphological Parameters of Solidago canadensis Root Systems

**Figure 3:**
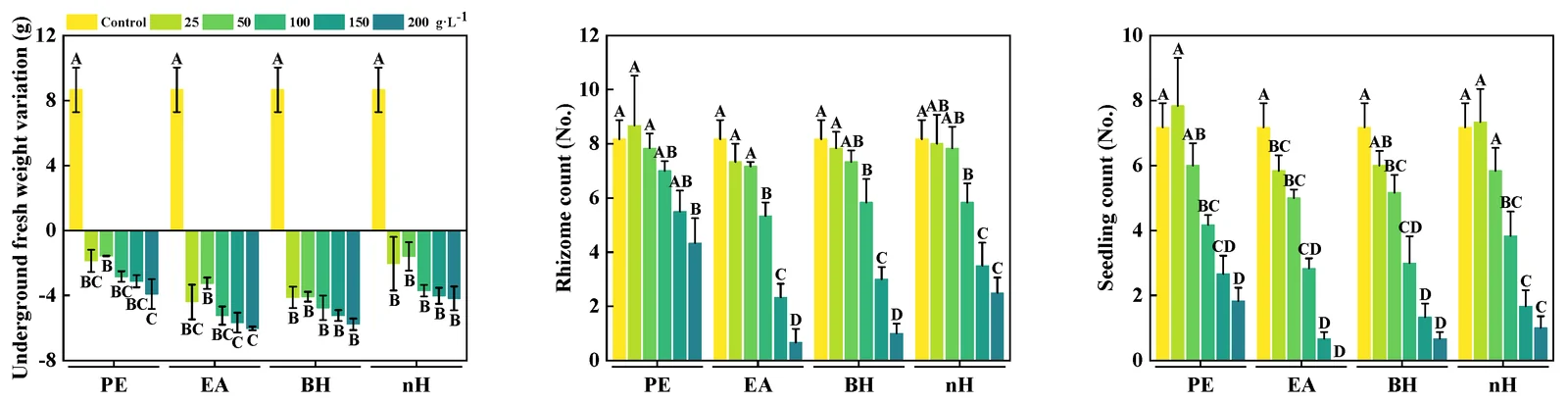
The Impact of *Humulus scandens* Leaf Extract on the Root System of *Solidago canadensis*. Different capital letters indicate statistically significant differences among concentration treatments (*p* < 0.01).

At equal concentrations, the four leaf extracts differed significantly in their effects on root morphology and biomass (Figure 3). Ethyl acetate extract exhibited the strongest inhibition, with the highest inhibition rates for underground fresh weight, rhizome count, and new seedling count, followed by n-butanol; n-hexane and petroleum ether showed relatively weaker activity. At 200 g·L^−1^, ethyl acetate extract reduced relative inhibitory effect index, rhizome count, and new seedling count by 169.6%, 91.8%, and 100%, respectively; n-butanol by 166.6%, 87.8%, and 90.7%; n-hexane by 148.3%, 69.4%, and 86.1%; and petroleum ether by 145.2%, 46.9%, and 74.4%.

Fresh weight change, rhizome number, and new seedling number decreased significantly with increasing extract concentration, indicating that the inhibitory effect intensified with concentration. Ethyl acetate treatment caused the largest reductions in underground fresh weight, resulting in respective reductions of 4.40 g, 3.24 g, 5.24 g, 5.67 g, and 6.02 g across treatment groups; rhizome and new seedling counts also declined progressively. At 200 g·L^−1^, ethyl acetate exerted the strongest effect on growth, with statistically significant differences compared with other concentrations. Overall, ethyl acetate was the most inhibitory among the four extracts.

### 3.4. Effects of Various Humulus scandens Leaf Extracts on the Physiological Parameters of Solidago canadensis

**Figure 4:**
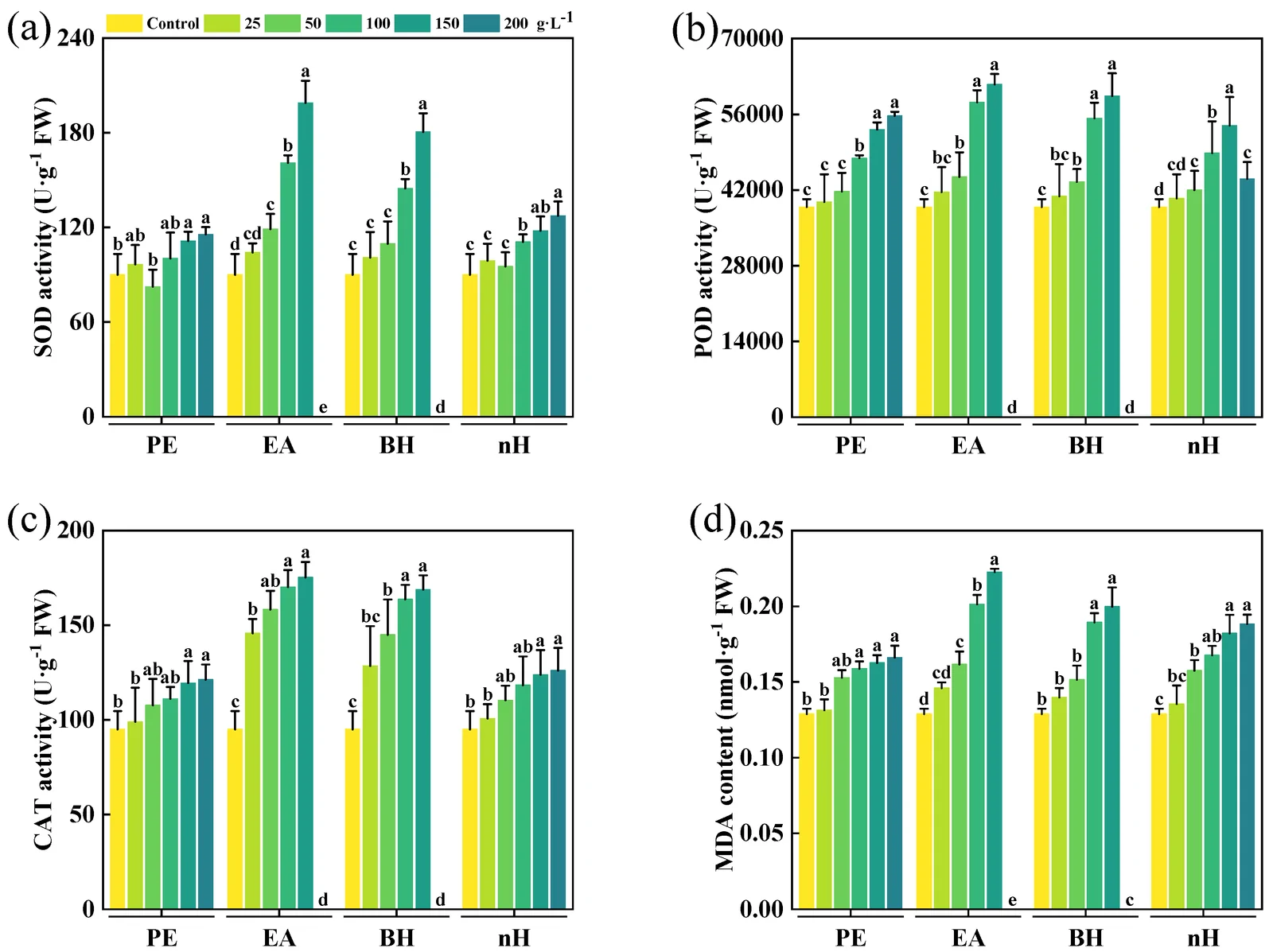
Effects of *Humulus scandens* Leaf Extract on the Physiological Indices of *Solidago canadensis* Roots. (**a**) SOD activity; (**b**) POD activity; (**c**) CAT activity; (**d**) MDA content. Different lowercase letters indicate statistically significant differences among concentration treatments (*p* < 0.05).

The leaf extracts of *Humulus scandens* exerted varying degrees of influence on the antioxidant enzyme system of the underground rhizomes of *Solidago canadensis*, including superoxide dismutase (SOD), peroxidase (POD), catalase (CAT), and the lipid peroxidation product malondialdehyde (MDA). All four extracts significantly altered SOD activity (Figure 4a). As the extract concentration increased, SOD activity in the rhizomes gradually rose, showing statistically significant differences compared to the control group. The maximum SOD activity was observed at a concentration of 150 g·L^−1^. In contrast, petroleum ether and n-hexane extracts at a concentration of 200 g·L^−1^ resulted in significantly higher SOD activity compared to the control group; however, at other concentrations, the effects of these two extracts on SOD activity were not statistically significant.

As the concentration of ethyl acetate and n-butanol extracts increased, CAT activity in the rhizomes exhibited a gradual upward trend (Figure 4c). At a concentration of 25 g·L^−1^, CAT activity was significantly higher than that in the control group and reached its peak at 150 g·L^−1^. In contrast, for the roots treated with petroleum ether and n-hexane extracts, CAT activity was significantly elevated compared to the control group at concentrations of 150 g·L^−1^ and 200 g·L^−1^; however, no statistically significant differences were observed at other concentrations.

POD activity increased with rising extract concentration (Figure 4b); however, for petroleum ether and n-hexane extracts, activity declined at a concentration of 200 g·L^−1^, while ethyl acetate and n-butanol extracts resulted in complete inactivation. Among all treatment groups, the MDA content was significantly higher than that in the control group, with the ethyl acetate extract exhibiting the most pronounced effect (Figure 4d). This suggests that treatment with the four extracts induced oxidative damage to the underground rhizomes of *Solidago canadensis* through allelopathic mechanisms.

### 3.5. Allelopathic Effects of Humulus scandens Leaf Extracts on Solidago canadensis Roots

The allelopathic effects of the four *H. scandens* leaf extracts on *S. canadensis* roots varied significantly with concentration (Table 2). For morphological parameters (underground fresh weight, rhizome number, and new seedling count), the allelopathic effect indices decreased progressively as extract concentration increased, indicating stronger inhibition. The comprehensive allelopathic effect index, which integrates all measured indicators, also declined in a concentration-dependent manner. In contrast, the allelopathic effect indices of seven physiological indicators related to protective enzyme activities increased with extract concentration, reflecting a stimulatory effect on root antioxidant systems.

Some individual indices at 25 g·L^−1^ were higher than at 50 g·L^−1^ (e.g., underground fresh weight and rhizome number under petroleum ether; underground fresh weight and comprehensive index under ethyl acetate and n-butanol; underground fresh weight and SOD activity under n-hexane). These non-monotonic fluctuations at low concentrations, however, did not alter the overall concentration-dependent trend. Across all concentrations, the inhibitory intensity followed the order: ethyl acetate > n-butanol > n-hexane > petroleum ether.

### 3.6. Comprehensive Analysis of Widely Targeted Metabolome Results of Humulus scandens Extract

**Figure 5:**
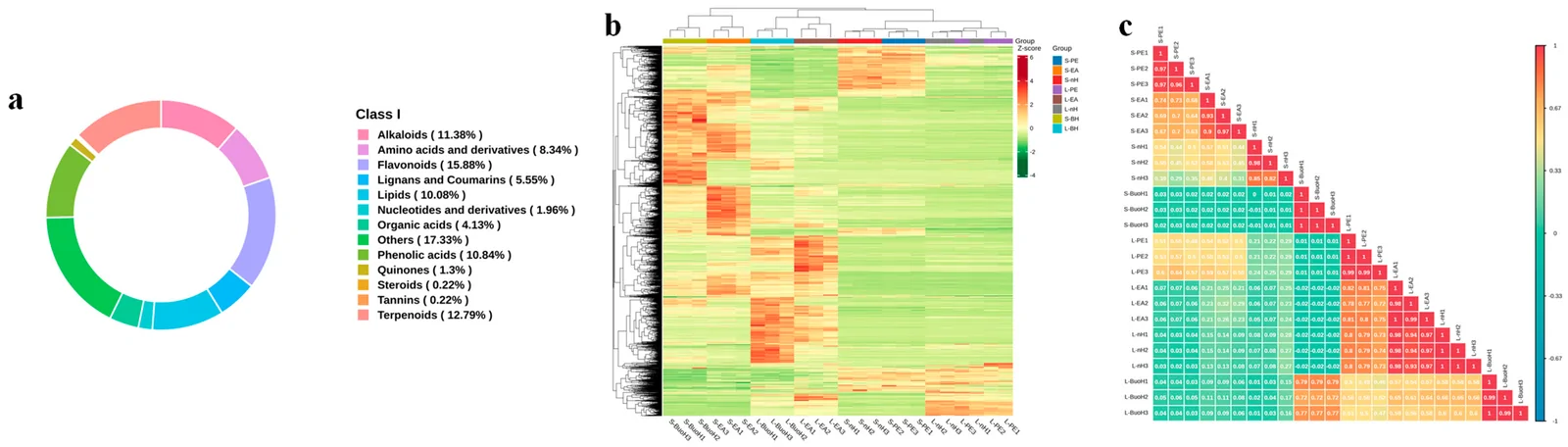
Metabolomics analysis of *Humulus scandens*. (**a**) Circular diagram illustrating the distribution of metabolite categories. (**b**) Hierarchical clustering heatmap depicting overall sample relationships. (**c**) Correlation heatmap showing inter-sample associations. Each treatment group included three biological replicates, as indicated by the numbers 1, 2, and 3 appended to the group names. Extract types include petroleum ether extract of stem (S-PE), ethyl acetate extract of stem (S-EA), n-butanol extract of stem (S-BH), n-hexane extract of stem (S-nH), petroleum ether extract of leaf (L-PE), ethyl acetate extract of leaf (L-EA), n-butanol extract of leaf (L-BH), and n-hexane extract of leaf (L-nH).

Through extensive targeted metabolomics analysis, a total of 2759 metabolites were identified in four extracts derived from the stems and leaves of *Humulus scandens*, which could be categorized into 13 distinct chemical classes (Figure 5a). The major categories included flavonoids, terpenoids, alkaloids, phenolic acids, and lipids, collectively accounting for 61.7% of the detected metabolites. Metabolite content data were subjected to unit variance scaling (UV scaling), and cluster heat map analysis was performed to evaluate the repeatability of the test samples. The results indicated significant differences among the various treatment groups, with high consistency observed across replicate experiments (Figure 5b,c).

### 3.7. PCA and OPLS-DA Analysis

**Figure 6:**
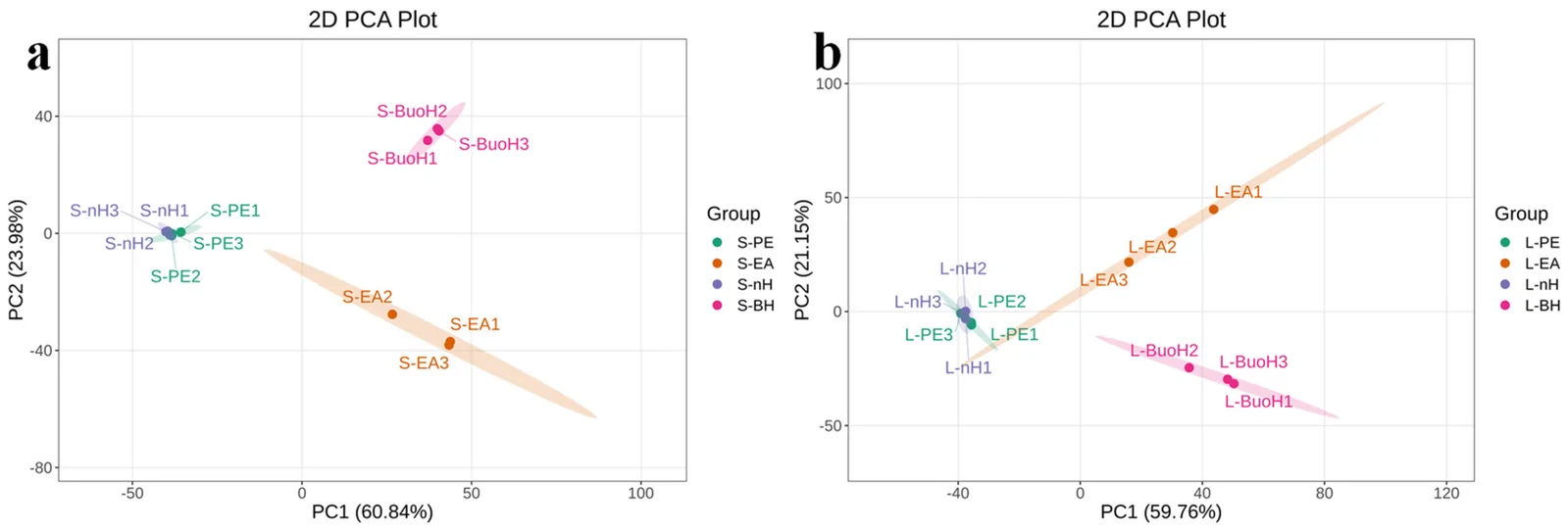
PCA of extracts from the stems and leaves of *Humulus scandens*. (**a**) Stem extract; (**b**) leaf extract. Each treatment group included three biological replicates, as indicated by the numbers 1, 2, and 3 appended to the group names. Extract types include petroleum ether extract of stem (S-PE), ethyl acetate extract of stem (S-EA), n-butanol extract of stem (S-BH), n-hexane extract of stem (S-nH), petroleum ether extract of leaf (L-PE), ethyl acetate extract of leaf (L-EA), n-butanol extract of leaf (L-BH), and n-hexane extract of leaf (L-nH).

To further investigate the differences in overall metabolite composition and intra-group sample variability among extracts from the stems and leaves of *Humulus scandens* prepared using four different solvents, PCA was performed. The PCA results (Figure 6) revealed that PC1 accounted for 60.84% of the variance in stem samples, while PC2 explained 23.98%. For leaf samples, PC1 explained 59.76% of the variance, and PC2 accounted for 21.15%. In terms of distribution patterns, samples within the same group exhibited strong clustering. However, partial overlap was observed between petroleum ether and n-hexane extracts, suggesting relatively minor metabolic differences between these two solvent systems. In contrast, the remaining sample groups displayed a clear separation, indicating significant differences in metabolite composition between the stems and leaves of *Humulus scandens*.

**Figure 7:**
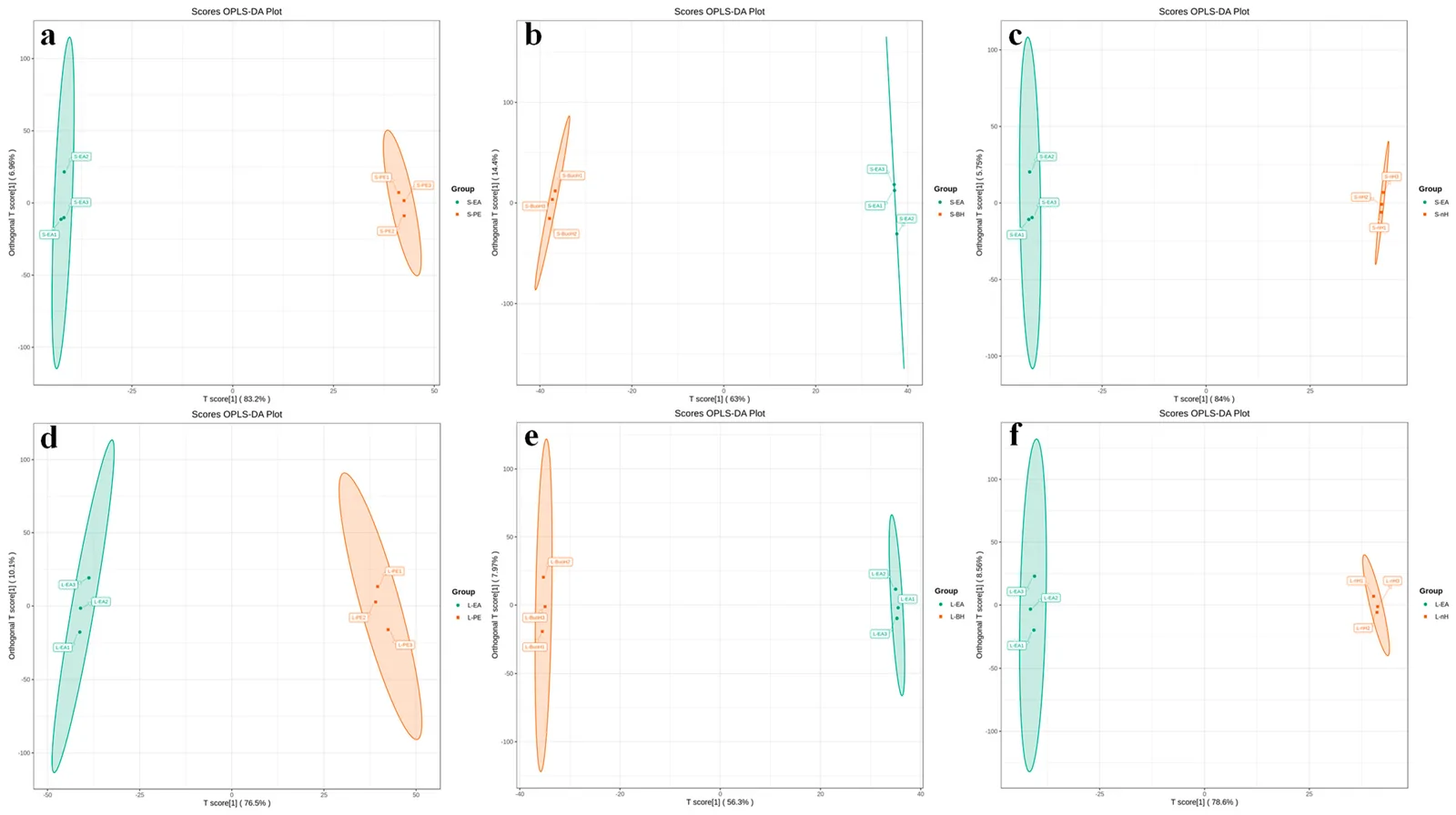
OPLS-DA score scatter plots. (**a**): S-EA vs. S-PE; (**b**): S-EA vs. S-BH; (**c**): S-EA vs. S-nH; (**d**): L-EA vs. L-PE; (**e**): L-EA vs. L-BH; (**f**): L-EA vs. L-nH. Each treatment group included three biological replicates, as indicated by the numbers 1, 2, and 3 appended to the group names. Extract types include petroleum ether extract of stem (S-PE), ethyl acetate extract of stem (S-EA), n-butanol extract of stem (S-BH), n-hexane extract of stem (S-nH), petroleum ether extract of leaf (L-PE), ethyl acetate extract of leaf (L-EA), n-butanol extract of leaf (L-BH), and n-hexane extract of leaf (L-nH).

In the allelopathic assays involving the germination of *Solidago canadensis* seeds and the growth of underground rhizomes, ethyl acetate was identified as the most allelopathically active fraction in the extract of *Humulus scandens*. To identify potential allelochemicals, comparative analyses were conducted between ethyl acetate and three other extraction solvents—petroleum ether, n-butanol, and n-hexane. In this study, an OPLS-DA model was employed to perform pairwise comparisons of samples extracted from the stems and leaves of *Humulus scandens* using different solvents (S-EA vs. S-PE; S-EA vs. S-BH; S-EA vs. S-nH; L-EA vs. L-PE; L-EA vs. L-BH; L-EA vs. L-nH) (Figure 7a–f). To assess model reliability, 200 permutation tests were carried out for each comparison group (Figure S1). The model exhibited strong predictive power, with Q^2^ values exceeding 0.9 across all comparisons, indicating high model stability and no evidence of overfitting. Therefore, the OPLS-DA model was deemed suitable for the identification of potential allelochemicals. The OPLS-DA score plot revealed a clear separation among different extracts, suggesting substantial differences in their chemical compositions.

### 3.8. Screening of Differentially Expressed Metabolites in Humulus scandens

**Figure 8:**
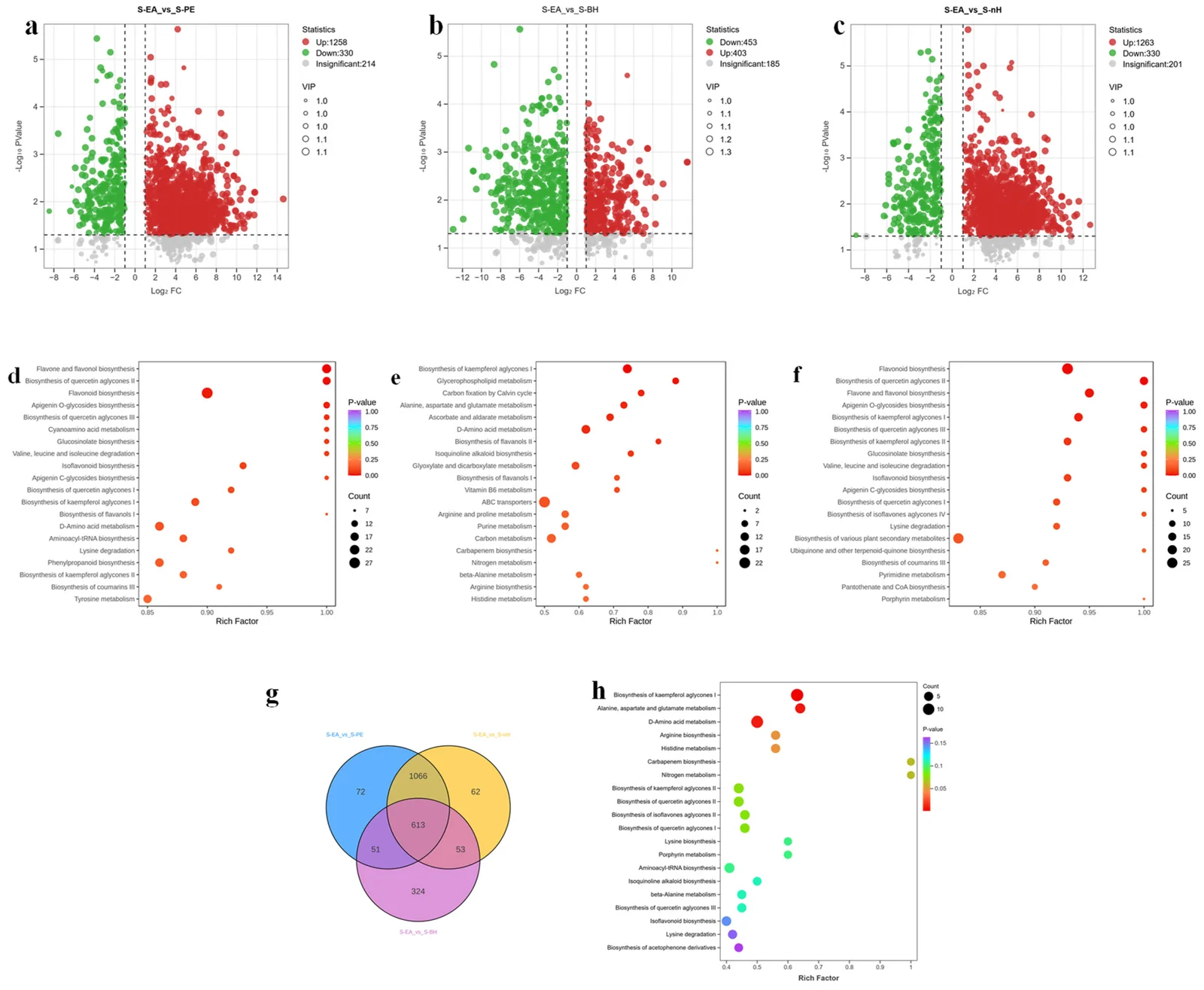
Screening and Metabolic Pathway Analysis of Differential Metabolites in the Stem Extract of *Humulus scandens.* (**a**–**c**) Volcano plots depicting differential metabolites ((**a**): S-EA vs. S-PE; (**b**): S-EA vs. S-BH; (**c**): S-EA vs. S-nH); (**d**–**f**) Pathway enrichment plots of differential metabolites (The top 20 pathways ranked by *p*-value, from smallest to largest, are shown. (**d**): S-EA vs. S-PE; (**e**): S-EA vs. S-BH; (**f**): S-EA vs. S-nH); (**g**): Venn diagram illustrating the overlapping and unique differential metabolites among the three group comparisons; (**h**): pathway enrichment map summarizing the metabolic pathways associated with the three group comparisons. In the volcano plots, green dots indicate down-regulated differential metabolites, red dots indicate up-regulated differential metabolites, and gray dots represent metabolites that were detected but did not show statistically significant differences. The size of each dot corresponds to the VIP value. In the pathway enrichment map, the color intensity reflects the significance of enrichment based on *p*-values, with redder colors indicating greater significance. The size of the dots represents the number of differential metabolites enriched in each pathway. Extract types include petroleum ether extract of stem (S-PE), ethyl acetate extract of stem (S-EA), n-butanol extract of stem (S-BH), and n-hexane extract of stem (S-nH).

Differential metabolites were identified using VIP ≥ 1, fold change ≥2 or ≤0.5, and *p* < 0.05. For stem extracts, the comparisons S-EA vs. S-PE, S-EA vs. S-BH, and S-EA vs. S-nH yielded 1588 (1258 up/330 down), 856 (403 up/453 down), and 1593 (1263 up/330 down) differential metabolites, respectively (Figure 8a–c). KEGG enrichment mainly involved flavonoid/flavonol biosynthesis, quercetin aglycone II biosynthesis, kaempferol aglycone I biosynthesis, glycerophospholipid metabolism, and carbon fixation in the Calvin cycle (Figure 8d–f). Venn analysis identified 613 common differential metabolites among the three comparisons, enriched in kaempferol aglycone I biosynthesis, alanine/aspartate/glutamate metabolism, D-amino acid metabolism, arginine biosynthesis, and histidine metabolism (Figure 8g,h; Supplementary Table S1).

**Figure 9:**
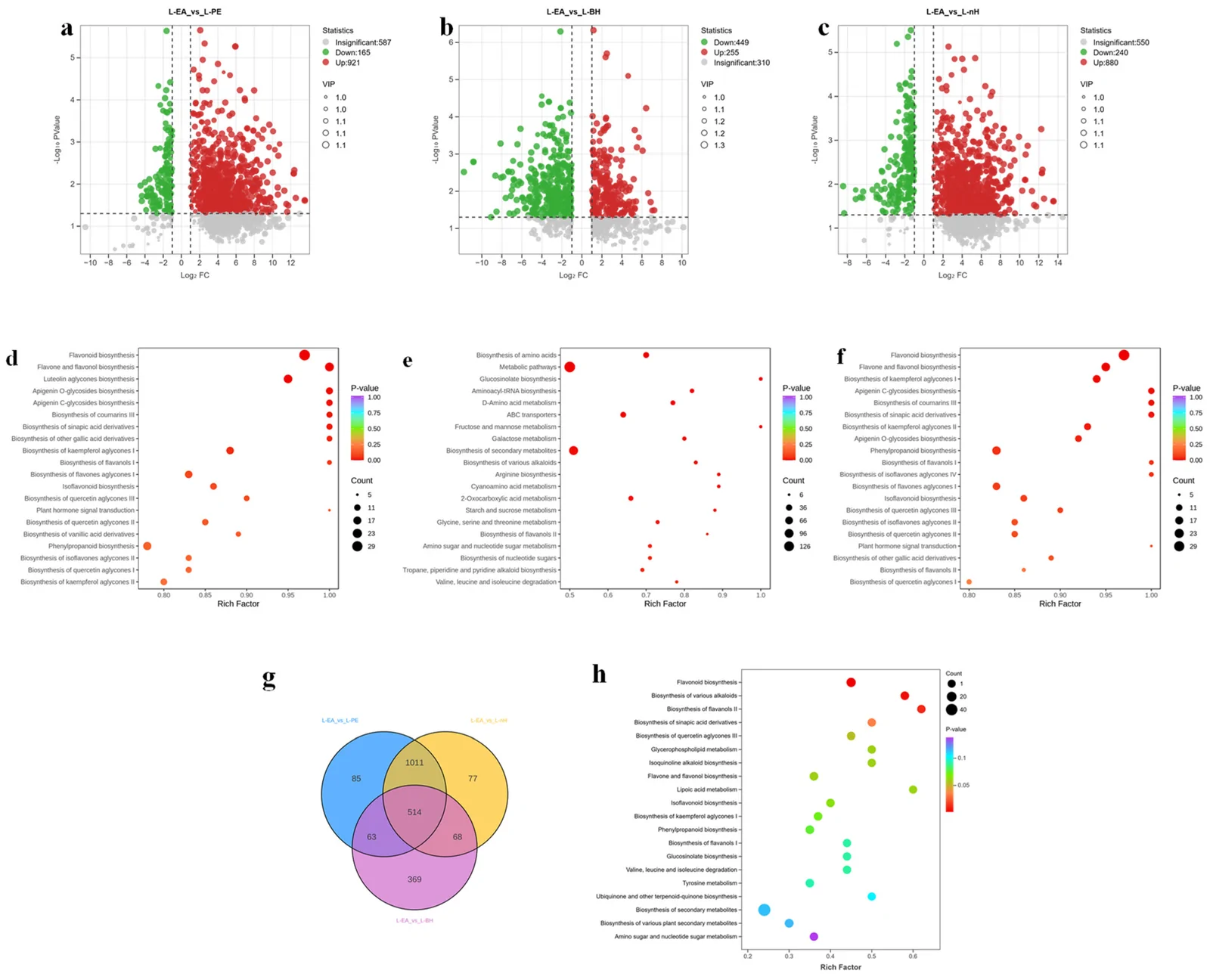
Screening and Metabolic Pathway Analysis of Differential Metabolites in the Leaf Extract of *Humulus scandens.* (**a**–**c**) Volcano plots depicting differential metabolites ((**a**): L-EA vs. L-PE; (**b**): L-EA vs. L-BH; (**c**): L-EA vs. L-nH); (**d**–**f**) pathway enrichment plots of differential metabolites (The top 20 pathways ranked by *p*-value, from smallest to largest, are shown; (**d**): L-EA vs. L-PE; (**e**): L-EA vs. L-BH; (**f**): L-EA vs. L-nH); (**g**): Venn diagram illustrating the overlapping differential metabolites among the three comparisons. (**h**): Pathway enrichment map summarizing the metabolic pathways associated with the three comparisons. In the volcano plots, green dots indicate down-regulated differential metabolites, red dots indicate up-regulated differential metabolites, and gray dots represent metabolites that were detected but not statistically significant. The size of each dot corresponds to the VIP value. In the pathway enrichment map, the color intensity reflects the significance of enrichment based on *p*-values, with redder colors indicating greater significance. The size of the dots represents the number of differential metabolites enriched in each pathway. Extract types include petroleum ether extract of leaf (L-PE), ethyl acetate extract of leaf (L-EA), n-butanol extract of leaf (L-BH), and n-hexane extract of leaf (L-nH).

For leaf extracts, the comparisons L-EA vs. L-PE, L-EA vs. L-BH, and L-EA vs. L-nH yielded 1086 (921 up/165 down), 704 (255 up/449 down), and 1120 (880 up/240 down) differential metabolites, respectively (Figure 9a–c). KEGG enrichment mainly involved flavonoid biosynthesis, flavonoid/flavonol biosynthesis, luteolin aglycone biosynthesis, amino acid biosynthesis, metabolic pathways, glucosinolate biosynthesis, and kaempferol aglycone I biosynthesis (Figure 9d–f). Venn analysis identified 514 common differential metabolites, enriched in flavonoid biosynthesis, biosynthesis of various alkaloids, flavanols II, sinapic acid derivatives, and quercetin aglycone III (Figure 9g,h; Supplementary Table S2).

**Figure 10:**
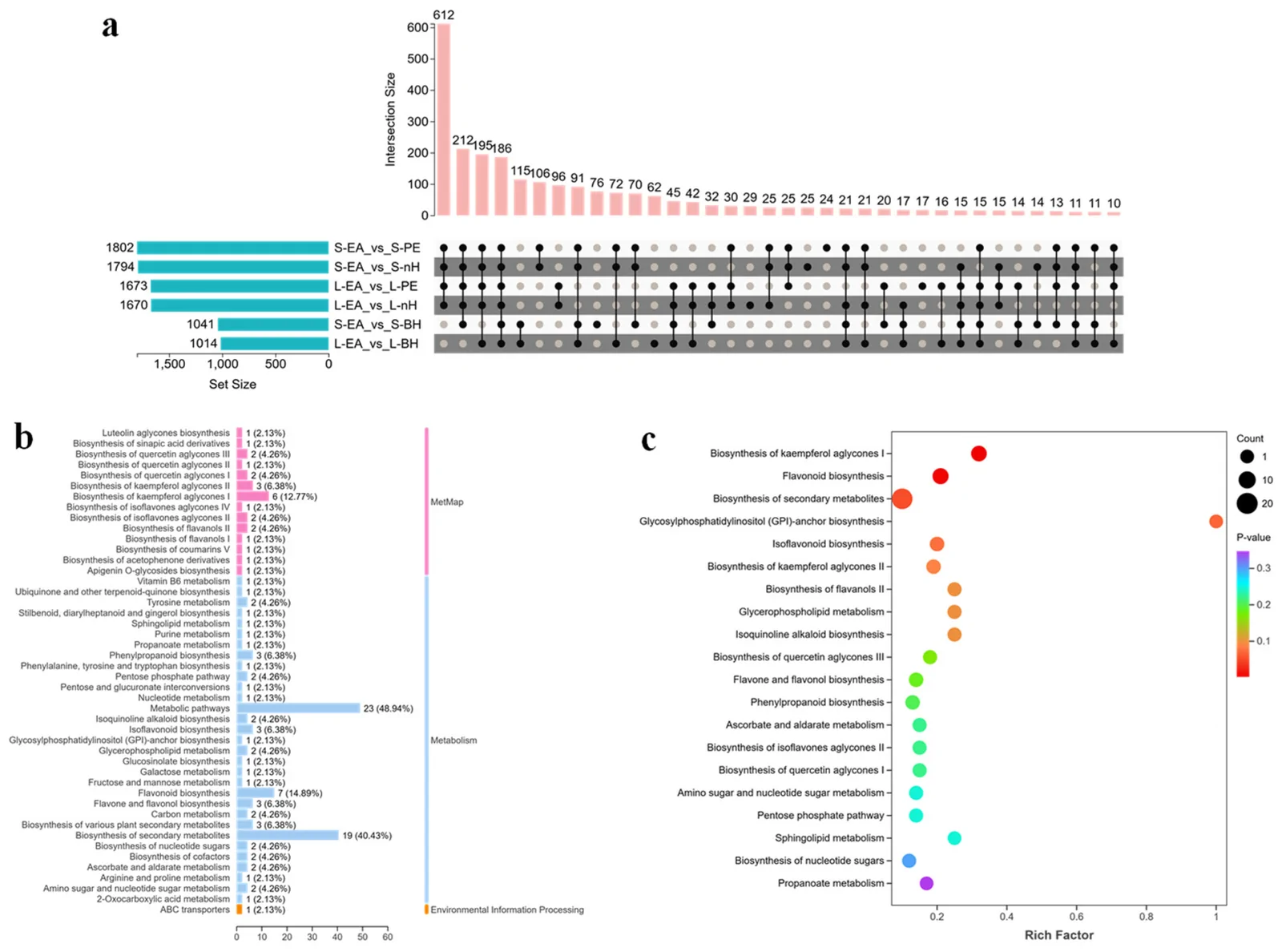
Differential Metabolite Screening and Metabolic Pathway Analysis Across Six Group Comparisons. (**a**): UpSet Plot depicting the overlap of up-regulated metabolites; (**b**): Classification of differential metabolite pathways; (**c**): Pathway enrichment analysis (the top 20 pathways ranked by *p*-value, from smallest to largest, are shown). In the enrichment map, the color intensity reflects the significance of the *p*-value, with redder colors indicating greater enrichment significance, and the size of the dots represents the number of differential metabolites enriched in each pathway.

Different extraction solvents significantly altered metabolite composition and pathway enrichment. UpSet analysis across the six comparisons revealed 186 commonly up-regulated compounds (Figure 10a; Supplementary Table S3). Enriched pathways were primarily associated with kaempferol aglycone I biosynthesis, flavonoid biosynthesis, biosynthesis of secondary metabolites, GPI-anchor biosynthesis, and isoflavonoid biosynthesis (Figure 10b,c), suggesting a key role for flavonoid metabolism in the allelopathic inhibition by ethyl acetate extract.

**Figure 11:**
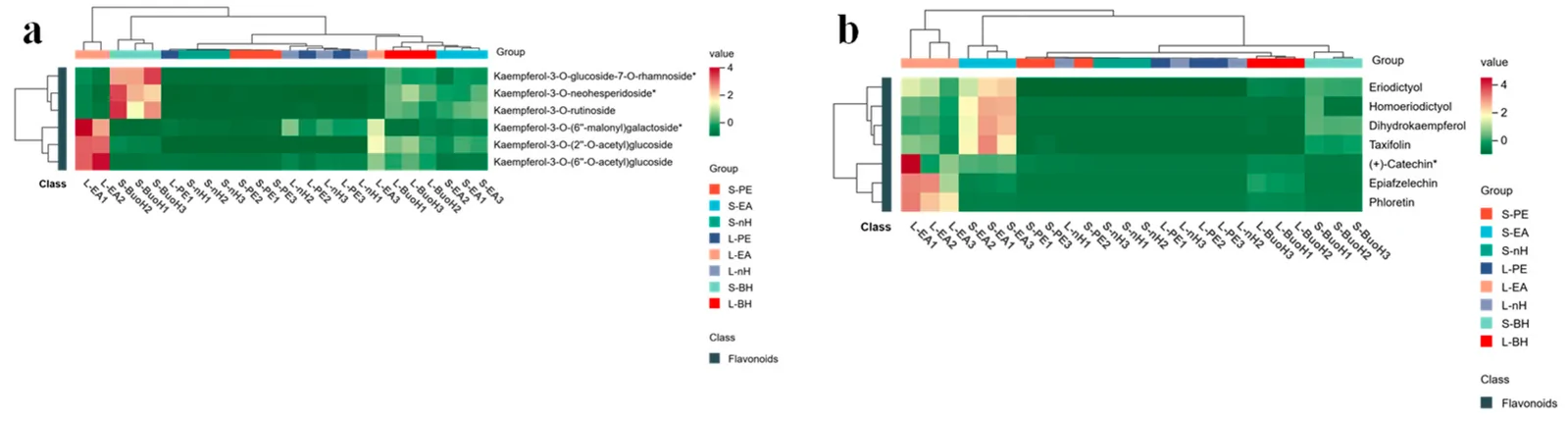

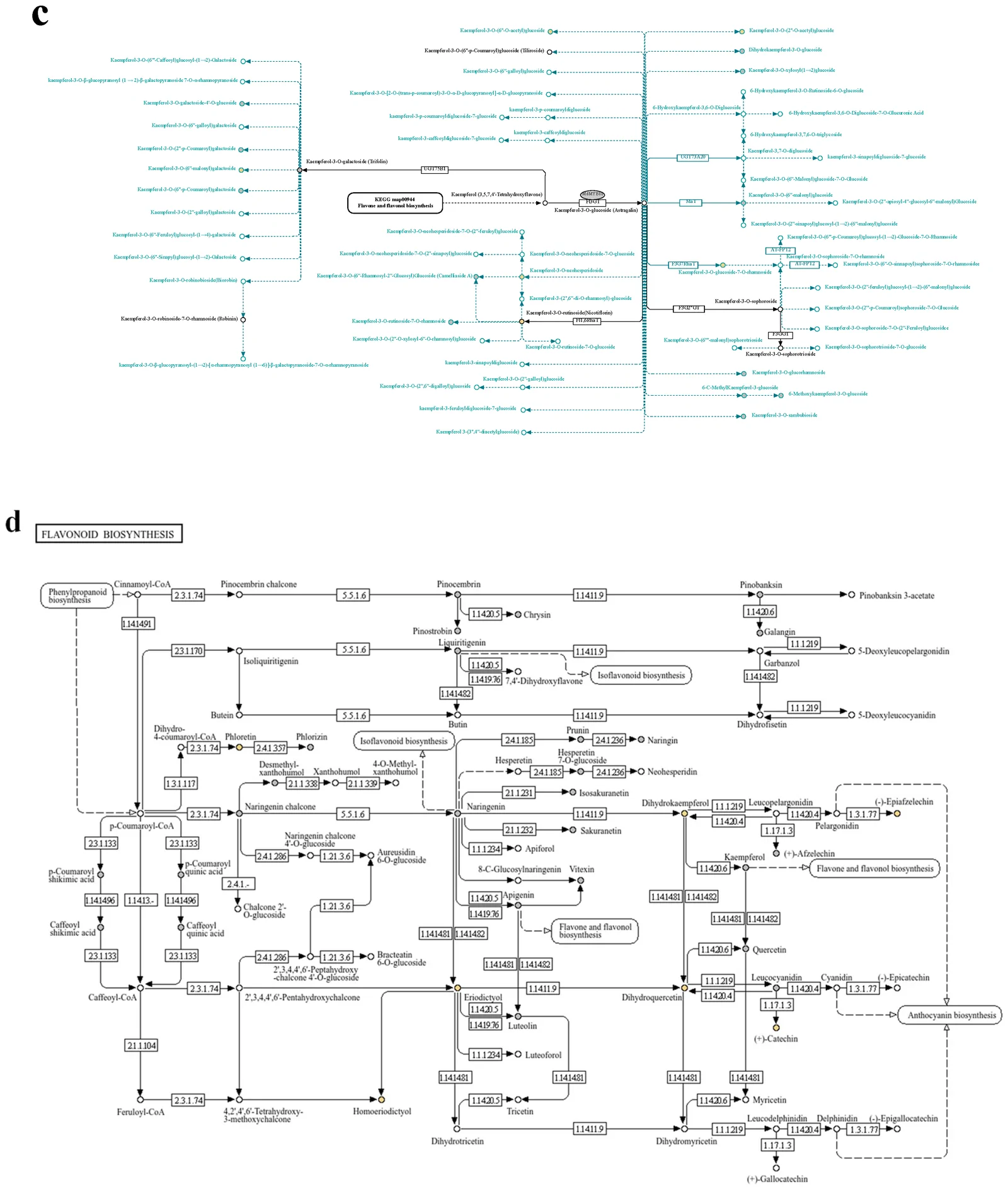
Heatmap and pathway map of differential metabolites. (**a**) Differential metabolites in the Biosynthesis of Kaempferol Aglycones I pathway (MetMap 113). (**b**) Differential metabolites in the Flavonoid Biosynthesis pathway (ko00941). (**c**) Representation of the Biosynthesis of Kaempferol Aglycones I pathway (MetMap 113). (**d**) Representation of the Flavonoid Biosynthesis pathway (ko00941). Red indicates higher metabolite content, while green indicates lower content. “*” indicates substances that have reached the highest classification level (Level 1). The circular nodes represent metabolites. Gray nodes indicate metabolites that were detected but showed no statistically significant change in abundance; and yellow nodes signify metabolites for which both upregulated and downregulated isoforms or derivatives were observed concurrently.

Cluster heatmap analysis identified six flavonoids in the kaempferol aglycone I pathway (MetMap 113) (Figure 11a,c) and seven in flavonoid biosynthesis (ko00941) (Figure 11b,d). Among these 13 flavonoids, 10 were up-regulated under ethyl acetate treatment, including kaempferol-3-O-(6″-malonyl)galactoside, kaempferol-3-O-(2″-O-acetyl)glucoside, kaempferol-3-O-(6″-O-acetyl)glucoside, eriodictyol, homoeriodictyol, dihydrokaempferol, taxifolin, (+)-catechin, epiafzelechin, and phloretin (Supplementary Table S4). These findings suggest that these 10 flavonoids may be key metabolites contributing to the allelopathic inhibitory effect of *H. scandens* ethyl acetate extract on *S. canadensis*.

## 4. Discussion

Allelopathy is a widespread phenomenon by which plant-derived chemicals can influence the germination and early growth of neighboring species [46]. While most studies have examined the allelopathic effects of invasive plants on native species, the reverse interaction—native plants inhibiting invasive species—may provide new insights and strategies for biological control [47]. In this study, seed germination and seedling growth of *Solidago canadensis* were used to evaluate the allelopathic potential of *Humulus scandens*. Germination and root elongation were inhibited in a concentration-dependent manner; high concentrations of both stem and leaf extracts caused significant inhibition, whereas low concentrations of leaf extract showed only mild inhibition and low concentrations of stem extract did not differ from the control. The radicle was more sensitive than the shoot, which was largely unaffected. These findings are consistent with reports that p-hydroxybenzoic acid from decomposing *Sorghum bicolor* straw inhibits radicle elongation without markedly affecting embryo growth in *S. canadensis* [48], and that *Lolium perenne* allelochemicals suppress root tip cell division in *Eupatorium adenophorum* seedlings but have minimal effects on embryonic development [49]. The greater sensitivity of the radicle may reflect its rapid cell division and elongation during early germination, which are particularly vulnerable to allelochemical exposure [50], and inhibition of embryonic respiration may further limit the ATP supply required for germination [51]. Allelopathic effects vary with plant species, extract source, and concentration [52,53]; although low concentrations sometimes stimulate germination [54,55], no such stimulation was observed here.

Extracts from different parts of *H. scandens* contain complex mixtures of allelochemicals with potential interactions [56], and their inhibitory intensity differed among organs, with leaf extracts being stronger than stem extracts. This is likely due to organ-specific differences in the types and concentrations of allelochemicals [57]. Leaves, as the primary photosynthetic organs, tend to accumulate high concentrations of defensive secondary metabolites, whereas stem allelochemicals may be stored in bound or less accessible forms requiring higher concentrations or specific conditions for release. Leaves are typically rich in phenolic acids and terpenoids, while stems may contain different alkaloid or flavonoid profiles [58,59], leading to distinct modes of action [60]. The observed differences may also involve multiple mechanisms such as disruption of antioxidant systems, reduced nutrient uptake, and interference with nucleic acid and protein synthesis [61]. Similar organ-dependent allelopathic effects have been reported for *Miscanthus sinensis* and *Sophora alopecuroides* [62].

Solvent polarity influences the types and polarities of extracted allelochemicals and thus the observed allelopathic activity [63]. In this study, all four solvent extracts inhibited seed germination and seedling growth, with the strongest effect from ethyl acetate, followed by n-butanol, n-hexane, and petroleum ether. This is consistent with the ability of moderately polar solvents such as ethyl acetate to efficiently extract phenolic acids, flavonoids, and certain terpenoids, which can disrupt cell membrane integrity, interfere with antioxidant systems, or modulate hormone signaling during germination [64]. Similar strong activity of ethyl acetate extracts has been reported for *Enteromorpha prolifera*, *Tridax procumbens*, and *Ligularia sagitta*. Non-polar solvents extracted less active components, and although n-butanol extracts many polar compounds, its effect may be attenuated by synergistic or antagonistic interactions among constituents [65]. Solvent polarity may also affect the environmental stability and bioavailability of allelochemicals; hydrophobic compounds extracted by ethyl acetate may adsorb onto soil particles and prolong activity, whereas more water-soluble or lipid-soluble components extracted by less polar solvents are more susceptible to leaching or microbial degradation [66].

Reactive oxygen species (ROS) are inevitable by-products of electron transport during plant energy metabolism [67,68]. Under allelochemical stress, excessive ROS accumulation can overwhelm cellular redox homeostasis, leading to lipid peroxidation, protein oxidation, and membrane damage [69,70]. Plants possess an antioxidant defense system comprising superoxide dismutase (SOD), peroxidase (POD), and catalase (CAT) to scavenge ROS and mitigate oxidative injury; malondialdehyde (MDA), a stable end-product of lipid peroxidation, is widely used as a biomarker of membrane impairment [71,72]. In the present study, the activities of SOD, POD, and CAT as well as the MDA content in the roots of *Solidago canadensis* increased gradually with increasing extract concentration. Among the four solvent extracts, the ethyl acetate extract induced the most pronounced enzymatic and lipid peroxidation responses, consistent with its efficiency in extracting phenolic acids, flavonoids, and other potential allelochemicals from *Humulus scandens* leaves [73]. These allelochemicals may act as pro-oxidants or interfere with mitochondrial electron transport, thereby triggering ROS overproduction and a compensatory upregulation of antioxidant enzymes [59,74]. However, the parallel accumulation of MDA and the occurrence of complete rhizome necrosis at 200 g·L^−1^ indicate that, at high allelochemical doses, ROS generation exceeded the scavenging capacity of the antioxidant system, resulting in severe oxidative damage. Such a dose-dependent pattern—activation of antioxidant defense at moderate stress but oxidative injury under severe stress—is characteristic of allelochemical-induced oxidative stress [17,75]. Therefore, oxidative damage is likely a key mechanism underlying the observed allelopathic inhibition, in agreement with previous findings in other allelopathic systems [76]. Further studies are needed to identify the specific ROS sources and signaling pathways activated by the major allelochemicals of *H. scandens* in *S. canadensis* rhizomes.

Metabolomic comparisons indicated that the chemical profiles of stem and leaf extracts differed substantially. Stem extracts contained higher numbers of alkaloids and amino acid derivatives, whereas leaf extracts were richer in terpenoids, lignans, and coumarins. These differences may reflect distinct ecological roles: volatile terpenoids may inhibit soil microbial activity [77], while alkaloids may interfere with signal transduction in receptor plants [78]. Many of the common up-regulated metabolites, particularly flavonoids, phenolic acids, and lignans/coumarins, were enriched in the ethyl acetate extract. This is consistent with the higher affinity of polar solvents for hydroxyl and carboxyl groups, which favors extraction of flavonoid glycosides and phenolic acids, whereas non-polar solvents such as petroleum ether preferentially extract terpenoids [79]. Allelopathic effects are generally not attributable to single compounds but rather to synergistic interactions among multiple allelochemicals through concentration addition, receptor synergy, or modulation of signal transduction pathways [80]. For example, a phenolic acid–flavonoid complex from *Populus* spp. root exudates inhibited *Echinochloa crusgalli* 2.3–4.7 times more strongly than individual components [81].

KEGG enrichment analysis identified the biosynthesis of kaempferol aglycones I and flavonoid biosynthesis as the main pathways associated with differentially expressed metabolites, with flavonoids up-regulated under ethyl acetate treatment. These flavonoids may be key contributors to the allelopathic inhibition of *S. canadensis*. Flavonoids are important secondary metabolites that enhance plant resistance to biotic and abiotic stresses and contribute to defense, environmental adaptation, and overall plant health [82]. Kaempferol glycosides such as kaempferol-3-O-glucoside and kaempferol-3-O-seabuckthornoside contribute to resistance against allelochemicals produced by invasive species [83], and their antioxidant properties help alleviate oxidative stress-induced cellular damage [84]. Accumulation of kaempferol and other flavonoids may also suppress germination and early growth of competing species, providing an ecological advantage [85].

As a native plant species, *Humulus scandens* exhibits a significant competitive advantage within the plant community due to its allelopathic properties. In contrast, *Solidago canadensis*, as an invasive species, suppresses the growth of native plants through rapid growth and intense resource competition. The leaf ethyl acetate extract of *Humulus scandens* exhibited the strongest allelopathic inhibition on *Solidago canadensis* at 200 g·L^−1^, providing a basis for developing targeted control techniques. This extract or *H. scandens* biomass could be applied locally as a bioherbicide or green mulch in invaded patches to suppress seed germination and rhizome regeneration, in line with allelopathy-based invasive plant management strategies [86,87].

## 5. Conclusions

The leaf extract of *Humulus scandens* exhibited stronger allelopathic inhibition on *Solidago canadensis* than the stem extract, with the ethyl acetate extract showing the most pronounced activity. This extract significantly suppressed seed germination and rhizome sprouting and disrupted the antioxidant enzyme system of the receptor plant. Metabolomic analysis identified ten flavonoids that were significantly upregulated in the ethyl acetate extract, predominantly enriched in the kaempferol aglycone biosynthesis I pathway (MetMap 113) and the flavonoid biosynthesis pathway (ko00941), suggesting that these compounds may play a key role in the observed allelopathic inhibition. These findings provide a theoretical basis for the biological control of *S. canadensis* through the utilization of *H. scandens* allelopathic resources.

## Figures and Tables

**Table 1:** Allelopathic Response Index of Morphological Indicators of *Solidago canadensis* Seeds to the Extract of *Humulus scandens*.

Type	Concentration /g·L^−1^	Stem					Leaf				
		Germination Rate	Germination Index	Bud Length	Root Length	SE	Germination Rate	Germination Index	Bud Length	Root Length	SE
PE	25	−0.88	−0.28	0.09	−0.03	−0.28	−0.41	−0.40	−0.12	−0.44	−0.34
	50	−0.98	−0.10	0.03	−0.08	−0.28	−0.53	−0.61	−0.20	−0.85	−0.55
	75	−1.00	−0.25	−0.12	−0.15	−0.38	−0.93	−0.96	−0.11	−0.90	−0.72
	100	−1.00	−0.47	−0.18	−0.19	−0.46	−0.95	−0.97	−0.17	−0.91	−0.75
	200	−1.00	−0.67	−0.17	−0.39	−0.56	−1.00	−1.00	−1.00	−1.00	−1.00
EA	25	−0.50	−0.61	−0.28	−0.58	−0.49	−0.88	−0.96	−0.30	−0.76	−0.72
	50	−0.72	−0.94	−0.30	−0.85	−0.70	−0.98	−0.99	−0.38	−0.90	−0.81
	75	−0.97	−0.94	−0.29	−0.75	−0.74	−1.00	−1.00	−1.00	−1.00	−1.00
	100	−0.97	−0.97	−0.33	−0.73	−0.75	−1.00	−1.00	−1.00	−1.00	−1.00
	200	−1.00	−1.00	−1.00	−1.00	−1.00	−1.00	−1.00	−1.00	−1.00	−1.00
BH	25	−0.31	−0.40	−0.16	−0.08	−0.24	−0.52	−0.83	−0.24	−0.60	−0.55
	50	−0.24	−0.32	−0.13	−0.05	−0.19	−0.78	−0.90	−0.17	−0.87	−0.68
	75	−0.29	−0.39	−0.17	−0.38	−0.31	−0.98	−0.99	−0.27	−1.00	−0.81
	100	−0.43	−0.55	−0.17	−0.26	−0.35	−1.00	−1.00	−1.00	−1.00	−1.00
	200	−0.72	−0.80	−0.15	−1.00	−0.67	−1.00	−1.00	−1.00	−1.00	−1.00
nH	25	−0.38	−0.38	−0.11	−0.05	−0.23	−0.50	−0.78	−0.13	−0.47	−0.47
	50	−0.28	−0.32	−0.08	−0.01	−0.17	−0.72	−0.82	−0.15	−0.88	−0.65
	75	−0.38	−0.44	−0.17	−0.03	−0.25	−0.97	−0.98	−0.17	−0.93	−0.76
	100	−0.48	−0.54	−0.17	−0.22	−0.36	−0.97	−0.98	−0.50	−0.99	−0.86
	200	−0.74	−0.81	−0.31	−0.34	−0.55	−1.00	−1.00	−1.00	−1.00	−1.00

**Table 2:** Allelopathic Response Index of Underground Rhizomes of *Solidago canadensis* to Leaf Extracts of *Humulus scandens*.

Type	Concentration /g·L^−1^	Fresh Weight Change of Underground Part	Rhizome Number	Number of Newborn Seedlings	SOD	POD	CAT	MDA	SE
Petroleum ether	25	−1.22	0.06	0.09	0.07	0.02	0.04	0.02	−0.13
	50	−1.18	−0.04	−0.16	−0.08	0.07	0.12	0.16	−0.16
	75	−1.28	−0.08	−0.28	0.02	0.12	0.13	0.17	−0.17
	100	−1.33	−0.14	−0.42	0.10	0.19	0.15	0.19	−0.18
	200	−1.45	−0.47	−0.74	0.22	0.30	0.22	0.23	−0.24
Ethyl acetate	25	−1.51	−0.10	−0.19	0.14	0.07	0.35	0.12	−0.16
	50	−1.37	−0.12	−0.30	0.24	0.13	0.40	0.20	−0.12
	75	−1.58	−0.29	−0.47	0.33	0.25	0.41	0.29	−0.15
	100	−1.61	−0.35	−0.60	0.44	0.33	0.44	0.36	−0.14
	200	−1.70	−0.92	−1.00	−1.00	−1.00	−1.00	−1.00	−1.09
n-butanol	25	−1.48	−0.04	−0.16	0.11	0.05	0.26	0.08	−0.17
	50	−1.47	−0.10	−0.28	0.18	0.11	0.35	0.15	−0.15
	75	−1.54	−0.24	−0.49	0.27	0.20	0.39	0.25	−0.17
	100	−1.55	−0.29	−0.58	0.38	0.30	0.42	0.32	−0.14
	200	−1.67	−0.88	−0.91	−1.00	−1.00	−1.00	−1.00	−1.06
n-hexane	25	−1.24	−0.02	0.02	0.09	0.04	0.06	0.05	−0.14
	50	−1.18	−0.04	−0.19	0.06	0.08	0.14	0.18	−0.14
	75	−1.34	−0.12	−0.28	0.12	0.14	0.18	0.19	−0.16
	100	−1.43	−0.29	−0.47	0.19	0.20	0.20	0.23	−0.19
	200	−1.48	−0.69	−0.86	0.29	0.12	0.25	0.32	−0.29

## Data Availability

The data presented in this study are available on request from the corresponding author.

## Supplementary Materials

The following supporting information can be downloaded at: https://www.mdpi.com/article/10.3390/life16091452/s1. Figure S1: Validation of the OPLS-DA Model; Table S1: List of 613 common differential metabolites from three stem extract comparisons and associated KEGG pathway enrichment; Table S2: List of 514 common differential metabolites from three leaf extract comparisons and associated KEGG pathway enrichment; Table S3: List of 186 common up-regulated metabolites across the six comparisons of stem and leaf extracts and associated KEGG pathway enrichment; Table S4: Up-regulated flavonoids in the kaempferol aglycone I pathway (MetMap 113) and flavonoid biosynthesis (ko00941) identified by cluster heatmap analysis under ethyl acetate treatment of H. scandens extract.
