# Supplementary material for: Allelopathic Effect of Humulus scandens Stem and Leaf Extract on Solidago canadensis
Source: Life (Basel). 2026 Aug 31;16(9):1452. doi: 10.3390/life16091452 (PMC13608287; doi:10.3390/life16091452)
Supplement: Supplementary file 1 [file life-16-01452-s001.zip › Figure S1.pdf]

Supplementary Materials

Title:

Allelopathic Effect of *Humulus scandens* Stem and Leaf Extract on *Solidago canadensis*

Authors:

Bo Xie, Yiming Lai, Jun Wang, Huijuan Cao, Sheng Qiang, Zhen Zhang, Zheng Zhang, Ming Tang, Chunhuo Zhou*, Zunkang Zhao*

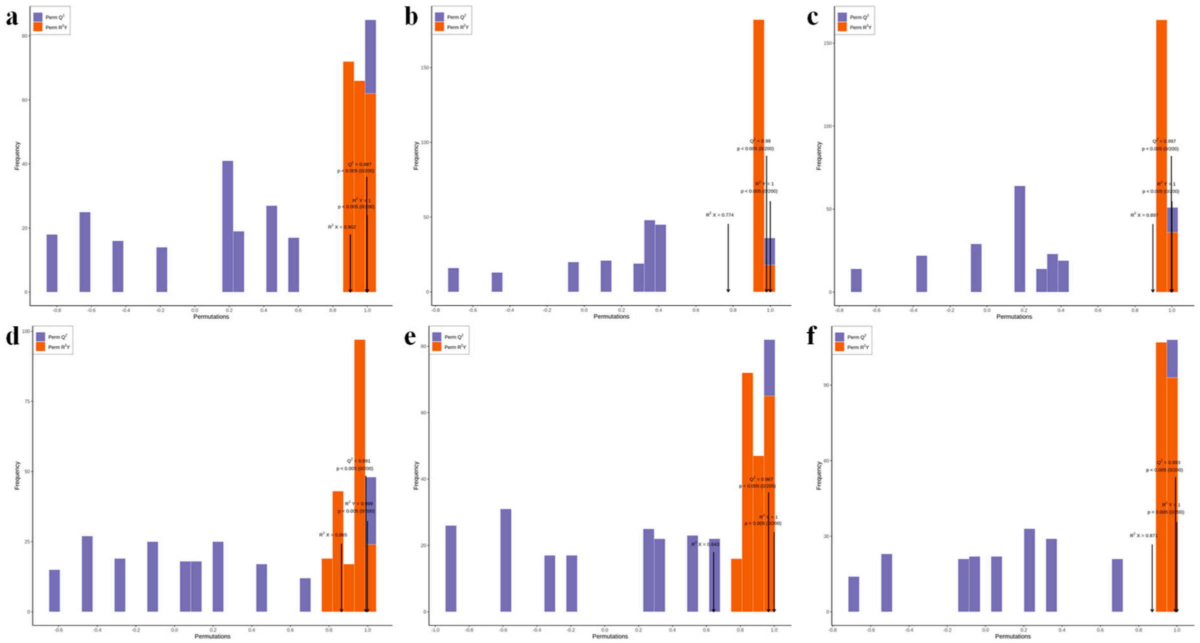

Figure S1. Validation of the OPLS-DA Model.

a: S-EA vs. S-PE; b: S-EA vs. S-BH; c: S-EA vs. S-nH; d: L-EA vs. L-PE; e: L-EA vs. L-BH; f: L-EA vs. L-nH. Extract types include petroleum ether extract of stem (S-PE), ethyl acetate extract of stem (S-EA), n-butanol extract of stem (S-BH), n-hexane extract of stem (S-nH), petroleum ether extract of leaf (L-PE), ethyl acetate extract of leaf (L-EA), n-butanol extract of leaf (L-BH), and n-hexane extract of leaf (L-nH).
